# The Impact of Infectious Disease-Related Public Health Emergencies on Suicide, Suicidal Behavior, and Suicidal Thoughts: A Systematic Review

**DOI:** 10.1027/0227-5910/a000753

**Published:** 2020-10-16

**Authors:** Tiago C. Zortea, Connor T. A. Brenna, Mary Joyce, Heather McClelland, Marisa Tippett, Maxwell M. Tran, Ella Arensman, Paul Corcoran, Simon Hatcher, Marnin J. Heise, Paul Links, Rory C. O'Connor, Nicole E. Edgar, Yevin Cha, Giuseppe Guaiana, Eileen Williamson, Mark Sinyor, Stephen Platt

**Affiliations:** ^1^ Suicidal Behaviour Research Laboratory, University of Glasgow, UK; ^2^ Department of Medicine, University of Toronto, ON, Canada; ^3^ National Suicide Research Foundation, Cork, Ireland; ^4^ Western Libraries, University of Western Ontario, London, ON, Canada; ^5^ School of Public Health, University College Cork, Ireland; ^6^ Ottawa Hospital Research Institute, Ottawa, ON, Canada; ^7^ Departments of Psychiatry and of Epidemiology and Biostatistics, Schulich School of Medicine and Dentistry, University of Western Ontario, London, ON, Canada; ^8^ Lawson Health Research Institute, London, ON, Canada; ^9^ Department of Psychiatry and Behavioural Neurosciences, McMaster University, Hamilton, ON, Canada; ^10^ Department of Psychiatry, University of Toronto, ON, Canada; ^11^ Usher Institute, University of Edinburgh, UK

**Keywords:** pandemics, epidemics, COVID-19, suicide, self-harm

## Abstract

**Abstract.** *Background:* Infectious disease-related public health emergencies (epidemics) may increase suicide risk, and high-quality evidence is needed to guide an international response. *Aims:* We investigated the potential impacts of epidemics on suicide-related outcomes. *Method:* We searched MEDLINE, EMBASE, PsycInfo, CINAHL, Scopus, Web of Science, PsyArXiv, medRxiv, and bioRxiv from inception to May 13–16, 2020. Inclusion criteria: primary studies, reviews, and meta-analyses; reporting the impact of epidemics; with a primary outcome of suicide, suicidal behavior, suicidal ideation, and/or self-harm. Exclusion criteria: not concerned with suicide-related outcomes; not suitable for data extraction. PROSPERO registration: #CRD42020187013. *Results:* Eight primary papers were included, examining the effects of five epidemics on suicide-related outcomes. There was evidence of increased suicide rates among older adults during SARS and in the year following the epidemic (possibly motivated by social disconnectedness, fears of virus infection, and concern about burdening others) and associations between SARS/Ebola exposure and increased suicide attempts. A preprint study reported associations between COVID-19 distress and past-month suicidal ideation. *Limitations:* Few studies have investigated the topic; these are of relatively low methodological quality. *Conclusion:* Findings support an association between previous epidemics and increased risk of suicide-related outcomes. Research is needed to investigate the impact of COVID-19 on suicide outcomes.

Suicide prevention is a crucial public health priority (World Health Organization, 2018). Key risk factors for suicide include psychological and social stressors, adverse life events, feeling trapped, life transitions and losses, physical illness, and mental disorders (Fazel & Runeson, 2020). Infectious disease-related public health emergencies are notable in that they simultaneously increase the presence and severity of multiple risk factors and, accordingly, some evidence suggests that they may have a greater impact on suicide rates than other catastrophic global events such as World Wars (Wasserman, 1992). It has therefore been hypothesized that the COVID-19 pandemic may increase suicide rates (Gunnell et al., 2020; Reger et al., 2020). It is also possible that an enhanced sense of belonging, resilience, and social connectedness or of finding meaning in the context of adversities like a global pandemic could potentially have the opposite impact, protecting against suicide (O'Connor & Kirtley, 2018; Reger et al., 2020).

Several specific features of the COVID-19 pandemic could contribute to suicide risk including prolonged quarantine, widespread societal fear, severe economic stress, medical equipment shortages, decreased access to mental healthcare, and the direct effect of coronavirus itself on the brain (Brooks et al., 2020; Gunnell et al., 2020; Reger et al., 2020; Rogers et al., 2020).

Specific populations such as young people, older adults, and frontline healthcare workers may be particularly vulnerable to the psychological impact of infectious outbreaks such as the COVID-19 pandemic (Kisely et al., 2020; Reger et al., 2020). However, these conjectures are as yet unconfirmed given the absence of relevant data. 

Expert recommendations propose increasing virtual connection for social support and healthcare delivery, increasing access to healthcare including evidence-based treatments for suicide-related variables, governmental financial safety nets, targeted means restriction interventions, and responsible media reporting that avoids stoking fear and hopelessness (Gunnell et al., 2020; Reger et al., 2020). Although this guidance is concordant with general recommendations for suicide prevention (Zalsman et al., 2016), specific recommendations should ideally incorporate evidence of the impact of pandemics on suicide and associated risk-reduction initiatives. There is therefore an urgent need for high-quality evidence to guide a proactive international response to the pandemic (Holmes et al., 2020).

The aim of this systematic review is to aggregate the existing evidence on the potential impact of infectious disease-related public health emergencies (referred to here as "epidemics") on suicide-related outcomes (for full list of research questions, see Electronic Supplementary Material 1 [ESM 1]). Given the anticipated dearth of research in this area, outcomes of interest include suicide death and related proxy outcomes, including nonfatal suicidal behavior, self-harm, and suicidal ideation. This review strives to achieve two key goals: (1) to facilitate dissemination of the best available knowledge to inform ongoing suicide prevention initiatives during the pandemic; (2) to identify key gaps in the literature to guide the research community in prioritizing studies that could have maximum impact on suicide prevention.

## Method 

### Protocol and Reporting Guidelines

The protocol for this systematic review was registered through PROSPERO (record ID CRD42020187013) and the Open Science Framework (https://osf.io/7hzu5/). We followed the Preferred Reporting Items for Systematic Reviews and Meta-Analysis (PRISMA) reporting guidelines for systematic reviews and the Synthesis Without Meta-Analysis (SWiM) complementary checklist.

### Search Strategy

Two research teams initially conducted independent searches (S1/S2 and S4) of the literature (see Figures A and B in ESM 1) before learning of one another's efforts and opting to consolidate into a single, unified consortium. A new search (S3), encompassing both teams' prior searches, was conducted and is referred to as the main search. A targeted gray literature search (S2) was also conducted on the impact of the COVID-19 pandemic on suicide-related outcomes. Initially, one of the teams carried out a complementary search of systematic reviews on HIV/AIDS outbreaks and suicide-related outcomes, but this was ultimately omitted from the review because it was determined that HIV/AIDS is fundamentally and mechanistically different from other, more rapidly spreading epidemics.

### Eligibility Criteria

For the main search, eligibility was determined with the following inclusion criteria: (1) peer-reviewed primary studies, reviews, and meta-analyses, of any design or type, (2) reporting the impact of any infectious disease-related public health emergency (exposures), with (3) a primary outcome of suicide, suicidal behavior, suicidal ideation, and/or self-harm (outcomes). There were no restrictions established for language or date of publication. Exclusion criteria were: (1) primary concern with broader mental health conditions and not suicide, (2) not reporting empirical findings suitable for data extraction (e.g., editorials, commentaries, book reviews, abstracts only), and (3) dissertations and theses.

The targeted gray literature search included any type of report from nonstandard sources (e.g., preprints) that were suitable for data extraction and focused on suicide-related outcomes in relation to the COVID-19 pandemic.

### Exposure and Outcomes

We followed the International Health Regulations (World Health Organization, 2016) definition of infectious disease-related public health emergencies: "An extraordinary event which is determined to constitute a public health risk to other states through the international spread of disease and to potentially require a coordinated international response," as it implies a "serious, sudden, unusual or unexpected" situation, which "carries implications for public health beyond the affected state's national border," and "may require immediate international action." 

Suicide-related outcomes encompassed suicide, attempted suicide, suicidal thoughts/ideation, and self-harm. Suicide was defined as an intentional, fatal, and self-harmful act undertaken with at least some intent to die; attempted suicide as an intentional, nonfatal, self-harmful act undertaken with at least some intent to die; suicidal thoughts/ideation as thoughts, considerations, or contemplation of suicide (including the desire to end one's own life or the presence of suicide plans/preparations); and self-harm as any intentional, nonfatal, self-harmful act, irrespective of motivation, intention, and method (although this review excludes the following behaviors from the category of self-harm: overeating, body tattooing and/or piercing, excessive consumption of alcohol or recreational drugs, starvation arising from anorexia nervosa, or unintentional harm to oneself).

### Information Sources

Literature searches were conducted using the appropriate subject headings and keywords across the following databases: MEDLINE, EMBASE, PsycInfo, CINAHL, Scopus, and Web of Science. The searches were conducted between May 13 and 16, 2020 by three members of the research team (TZ, MT, CB). The gray literature search was conducted in the preprint platforms PsyArXiv, medRxiv, and bioRxiv on May 16, 2020. Full search strategies for all searches are available in ESM 1.

### Study Selection

Duplicate studies identified by our main search were cross-checked and removed, publication titles and abstracts were independently screened by six reviewers (CB, MMT, PC, NEE, HMc, MJ), and full-text publications were independently assessed by six reviewers (EA, RCOC, PL, YC, SH, NEE) to determine suitability for inclusion. Ultimately, every abstract and full text was independently screened by two reviewers, and disagreements were resolved by a third reviewer (CB). Inter-rater reliability analysis for both screening stages can be found in the ESM 1.

### Data Collection Process

A proforma data extraction tool (available in ESM 1) was developed based on tools employed in previous systematic reviews (Zortea et al., 2019) and adapted to fit the research questions of this present review. For each study evaluated, the following data were extracted: study characteristics and design, sample characteristics, measures/description of suicide-related outcomes, measures/description of infectious disease-related public health emergencies, and results. Information on the mechanisms and processes between exposure and condition was extracted where possible.

### Quality Assessment

A quality assessment framework was adapted from previous reviews (O'Connor et al., 2016; available in ESM 1) and used to assess research design, measurement of suicide-related outcomes, relationship between infectious disease-related public health emergencies and outcomes, study sample, and whether the analyses were sufficiently powered (where power is relevant). To limit bias, three members of the research team cross-checked the quality assessment of each article (MJ, PL, TZ).

### Data Synthesis and Narrative Review

Given heterogeneity in study designs, populations, measures, constructs, and infectious disease-related public health emergencies, it was not feasible to conduct a meta-analysis. We therefore chose to employ a narrative synthesis approach. We extracted qualitative and quantitative data according to the themes of our research questions and, mindful of distinctions in both psychological processes and consequences underpinning different modes of suicide-related outcomes (Silverman, 2006), we organized our findings according to their relevance to suicide deaths and to nonfatal suicide-related behavior.

## Results 

**Figure 1:**
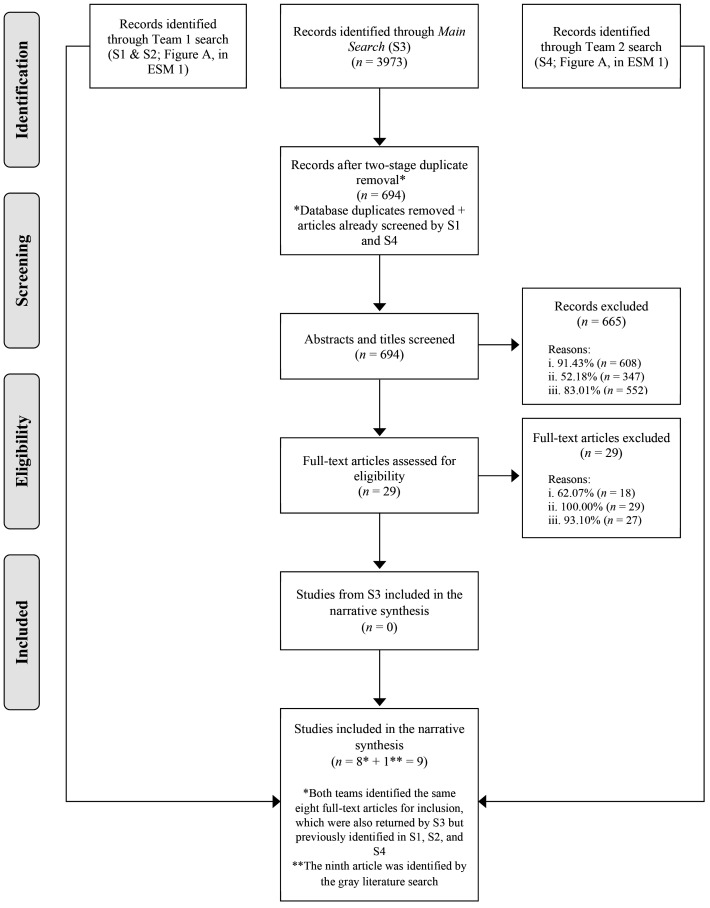
PRISMA diagram for main search (S3).Preferred reporting items for systematic reviews and meta-analyses (PRISMA) flow diagram. Reasons for exclusion: (i) the publication did not focus on suicidal behavior, suicidal ideation, and/or self-harm; (ii) the publication did not focus on any infectious disease-related public health emergency; (iii) the publication was excluded due to its publication type (not a primary study, systematic review, or meta-analysis); (iv) the publication did not contain empirical data eligible for data extraction and quality assessment; and (v) the publication was a duplicate of another in the study pool, missed in the initial duplicate removal.

Collectively, the original study teams' searches (see Figures A and B in ESM 1) and consortium main search (Figure 1) identified a combined total of 8,413 titles and abstracts from three unique search strategies across all six databases. From this original pool, 3,583 titles and abstracts were screened, 84 advanced to full-text screening, and eight primary articles plus one preprint were ultimately selected for inclusion in the systematic review. The search also identified a small number of case reports (available in ESM 1). Our complementary search of gray literature yielded one preprint (Ammerman et al., 2020; publication under peer-review; details available in ESM 1).

The eight primary studies investigated the impact of epidemics on suicide-related outcomes. These papers were published between 1992 and 2017 and examined the effects of epidemics that occurred from 1889 to 2016, including the Great Influenza Epidemic (Wasserman, 1992), Russian influenza (Honigsbaum, 2010), Severe Acute Respiratory Syndrome (SARS; Chan et al., 2006; Cheung et al., 2008; Huang et al., 2005; Yip et al., 2010), and Ebola virus disease (EVD; Keita et al., 2017). Five of these studies focused on suicide deaths (Chan et al., 2006; Cheung et al., 2008; Honigsbaum, 2010; Wasserman, 1992; Yip et al., 2010), two on attempted suicide (Huang et al., 2005; Okusaga et al., 2011), and one both on suicidal thoughts and attempts (Keita et al., 2017). These studies were conducted in Hong Kong, Taiwan, Guinea, the United States, and the United Kingdom, and employed naturalistic observational designs, analysis of historical archives, case-comparison matched by demographics, and cross-sectional case-control methodologies. Study characteristics are summarized in Table A1 in the appendix.

### Infectious Diseases-Related Public Health Emergencies and Suicide-Related Outcomes

#### Suicide Death

Only three of the five studies that focused on suicide deaths included primary data; all three examined the impact of SARS on suicide deaths (Chan et al., 2006; Cheung et al., 2008; Yip et al., 2010) in older adults (age ≥ 65). Chan and colleagues (2006) reported an increase in older-adult suicide rates in Hong Kong during the SARS epidemic in 2003 compared with the previous year (see Table A1 in the appendix). This increase reached statistical significance for women but not for men or for those younger than 65. The authors of the study hypothesized that a higher prevalence of preexisting risk factors for suicide (i.e., physical health problems, marginalized social support) in older adults may have increased their vulnerability to negative impacts of mandated social isolation and diminished access to non-emergency healthcare services. 

Cheung and colleagues (2008) conducted a more comprehensive study of the same period. Their analyses demonstrated associations between diminished social networking during the SARS epidemic with increasing rates of older adult suicides in Hong Kong in 2003, predominantly in April (1 month after the outbreak began) compared with previous reference years (1993, 1997, 1998, 2001, and 2002). This study also reported that older-adult suicide rates remained elevated above pre-epidemic outbreak rates in the first year following SARS. Older individuals with terminal or severe illness represented a smaller proportion of those who died by suicide in the post-SARS period. Elevated suicide rates were also reported before and during the epidemic for older adults who were totally or partially dependent. Among those who died by suicide, the proportion of older adults who were worried about becoming sick was substantially larger during the outbreak period.

In order to identify potential contributors to older adult suicide deaths associated with the SARS epidemic, Yip and colleagues (2010) investigated suicide notes and witnesses' descriptions of the deaths by suicide of 22 individuals for whom SARS was noted as a contributing factor. Older adults who died by suicide were more likely to be afraid of contracting SARS and to have fears of disconnection from others than non-SARS-related older-adult suicide decedents. Concern about burdening others was also identified as a motive for suicide. The authors reported no significant differences in sociodemographic factors, employment status, medical or psychiatric profiles, or level of dependence on others between these two groups.

An additional two studies examined the impact of the Great Influenza Epidemic on American suicide death rates between 1910 and 1920 (Wasserman, 1992) and the Russian influenza on regions of the United Kingdom at the end of the 19th century (1889–1893) (Honigsbaum, 2010), respectively. Wasserman (1992) found that, after controlling for indicators of alcohol prohibition, war, publicized suicide stories, and unemployment, the mortality rate variable (treated as an indicator of the impact of the epidemic) was positively associated with the suicide rate (estimated coefficient ordinary least squares and second-order autoregressive estimate 0.10, *p* < .05). In the second study, Honigsbaum (2010) examined historical data available from medical officers of health and national and local newspaper reports in the United Kingdom, as well as the poetry and memoirs of prominent survivors. The findings suggested that the epidemic coincided with a marked rise in the suicide rate. Coroners' verdicts of suicide in England and Wales, of whom 60% were male, increased by 25% between 1889 and 1893, and in 1893 the suicide rate peaked at 8.5 per 100,000. No demographic differences were reported in either study.

#### Attempted Suicide and Suicidal Thoughts

The association between epidemics and attempted suicide was investigated in three studies (Huang et al., 2005; Keita et al., 2017; Okusaga et al., 2011). Huang and colleagues (2005) investigated the demographic and clinical characteristics of emergency department (ED) adult patients before, during, and after the SARS epidemic in a SARS-dedicated hospital in Taiwan. The authors reported a correlation between overdose-related suicide attempt ED presentations and the peak-epidemic stage compared with all other (pre-, early, late, and post-) epidemic stages. Keita and colleagues (2017) examined patients who survived Ebola in Guinea between 2015 and 2016 and who received a psychiatric consultation (*n* = 33). Three patients who had been diagnosed with severe depression attempted suicide within 12 months of being discharged from the Ebola Treatment Centre, and one patient reported suicidal ideation only. Okusaga and colleagues (2011) investigated associations between attempted suicide and seropositivity for influenza (A and B) and coronaviruses among adults and found that seropositivity for influenza B was significantly associated with a history of suicide attempt(s), but not in those who had been diagnosed with influenza A or coronaviruses. This study did not restrict its analysis to individuals contracting influenza in an epidemic context, nor did the design allow for a determination of whether infection preceded the suicide attempt.

## Discussion 

### Summary and Interpretations of Findings

To our knowledge, this is the first comprehensive systematic review examining the effects of epidemics on suicide-related outcomes. Following a rigorous search of all relevant global bibliographic databases, eight primary studies were included in our review, in addition to one preprint article and four case studies, demonstrating a paucity of literature in this area. These eight primary reports were of relatively low methodological quality (see Table A1 in the appendix), and most failed to report statistical power. Given challenges inherent in studying public health emergencies, including an absence of baseline data and lack of experimental controls, we cannot draw a firm conclusion regarding a direct relationship between epidemics and suicide-related outcomes.

Evidence exists, however, suggesting a possible impact of the 2003 SARS epidemic on suicide deaths among older adults in Hong Kong (Chan et al., 2006; Cheung et al., 2008; Yip et al., 2010), particularly for older women, individuals with more severe illnesses, and those who were totally or partially dependent upon others. Although of poorer quality, data from the Great Influenza Epidemic (Wasserman, 1992) and Russian influenza (1889–1893) also suggest an association with suicide deaths (Honigsbaum, 2010).

Ebola infection history and influenza B seropositivity were both associated with attempted suicide (Keita et al., 2017; Okusaga et al., 2011) and preprint data for the COVID-19 pandemic suggest increases in both suicidal thoughts and suicide attempts (Ammerman et al., 2020). Given the lack of baseline data in the study by Keita and colleagues (2017), however, a causal link between the EVD episode and attempted suicide cannot be assumed.

One of the research questions of the present systematic review was: What can we learn from previous infectious disease-related public health emergencies about the likely effectiveness of different interventions to mitigate the impact of the COVID-19 pandemic on suicide-related outcomes? The identified studies, however, did not examine the effectiveness of specific interventions in mitigating the impact of pandemics on the incidence of suicide-related outcomes. This highlights the urgent need for primary studies of such interventions in the context of the COVID-19 pandemic, in order to inform suicide prevention policies and clinical practice. In the meantime, national governments should strengthen their commitment to developing and implementing suicide prevention programs and activities for which there is established evidence of effectiveness (Platt & Niederkrotenthaler, 2020).

Despite methodological limitations of the studies included in the review, synthesis of the outcomes supports an association between previous infectious disease-related public health emergencies and increased risk of suicidal thoughts, behavior, and deaths. It remains to be seen whether a similar finding will emerge for COVID-19.

### Pathways From Public Health Emergencies to Suicide-Related Outcomes

Despite the limited available evidence, there are some lessons that can be learned from previous infectious disease-related public health emergencies about the potential impact of the COVID-19 pandemic on the incidence of suicidal behavior, suicidal thoughts, and self-harm.

Four of the studies included in this review provide some, albeit limited, insight into the possible pathways leading to suicide-related outcomes. Several psychosocial processes have been reported or hypothesized, including existential anxiety or "dread" associated with media reports during the late 19th century Russian influenza outbreak in the United Kingdom (Honigsbaum, 2010) and fear/worry of contracting the virus, pessimism, helplessness, isolation, loneliness, and disconnectedness linked to the SARS outbreak (Chan et al., 2006; Cheung et al., 2008; Yip et al., 2010). Drawing on the wider literature on public health emergencies and the key features of typical government responses to such emergencies (including quarantine/physical distancing/self-isolation, restrictions on movement, travel and social interaction, and enforced closure of nonessential workplaces, educational establishments, places of worship, and community meeting places), a more comprehensive list of likely negative psychosocial impacts in the exposed population can be developed. Among these constructs are several established risk factors for suicide-related outcomes, including depression, anxiety, posttraumatic stress disorder (PTSD), hopelessness, fear, unresolved anger, guilt, worthlessness, sleep problems, self-stigmatization, feelings of entrapment and burdensomeness, substance misuse, loneliness, social isolation, disconnectedness, disruption of everyday routines, unemployment, financial strain/insecurity, domestic violence, and child neglect/abuse (Cullen et al., 2020; Dong & Bouey, 2020; James et al., 2019; Jeste et al., 2020; Oyesanya et al., 2015; Reger et al., 2020). Since many of these factors are also associated with accessing medical assistance in dying (MAID; Castelli Dransart et al., 2019) it remains to be seen whether epidemics led to increases in MAID as well. Exploration of the psychiatric and neuropsychiatric presentations associated with severe coronavirus infections (Rogers et al., 2020; Tucci et al., 2017) highlights the frequent occurrence of delirium/confusion in the acute phase, and higher levels of depression, fatigue, insomnia, anxiety, and PTSD in the postacute phase. 

The potential suicidogenic impact of public health emergencies may be greater in certain population or professional groups, in particular older adults (Chan et al., 2006; Cheung et al., 2008; Reger et al., 2020; United Nations, 2020; World Health Organization, 2020; Yip et al., 2010), people who are or become unemployed or underemployed (Black Dog Institute, 2020; Yao et al., 2020), people with preexisting mental health and/or substance misuse problems (Black Dog Institute, 2020; Cullen et al., 2020; Yao et al., 2020), and frontline health and social care staff (Cullen et al., 2020; World Health Organization, 2020). In the absence of information on the timing of an increase in the incidence of suicide in previous infection-related public health emergencies, we have examined the evidence relating to public health emergencies arising from natural disasters. While there is no consistency in the findings over the longer term (Matsubayashi et al., 2013), there is some evidence to suggest a short-term decrease in suicide in the immediate aftermath of a disaster. This has been labelled the "honeymoon period" (Madianos & Evi, 2010) or the "pulling together" phenomenon (Gordon et al., 2011). It has been claimed (Wilkinson & Pickett, 2020) that, at least in the early acute phase of the current pandemic, social connectedness, community cohesion, mutual support, and caring have increased in the United Kingdom. Pulling together at scale, if actually present, would indeed be expected to moderate the negative psychosocial impact of COVID-19, including on suicide-related outcomes. Theory and research similarly support the role of enhancing social outreach, connection, support, and perceptions of meaning in life and of mattering to others when seeking to reduce risk for suicide ideation and behavior among older adults (Heisel & Flett, 2016; Van Orden et al., 2010); such efforts (e.g., telephone support, older befriending programs, and meaning-centered psychosocial groups) may be especially needed to help counteract the potentially negative impact of epidemics on suicide risk in later life (De Leo et al., 2002; Heisel et al., 2020).

## Strengths, Limitations, and Gaps 

This timely review has a number of strengths, including the comprehensive list of databases and articles screened and the inclusion of a broad range of study outcomes of potential relevance to the relationship between pandemics and suicide.

The review also has a number of limitations, including a limited pool of studies and a mean quality assessment score of 3.9 out of 9 across all eight included articles. The research summarized in this review was conducted in multiple countries across different periods, cultures, and socioeconomic conditions. Although this represents the best available evidence to date, the degree to which it is generalizable to the current pandemic is unclear. In particular, the recent proliferation of both social distancing as well as virtual meeting technology introduces novel variables that could help to exacerbate and/or mitigate harms with no data, as yet, to quantify any such effects. Finally, the most important limitation of the review is that it relies on an existing evidence base that is exceedingly limited, particularly when considered in view of the potential impact of the COVID-19 pandemic on mental health and suicide worldwide. For these reasons, the data presented here must be interpreted with a strong note of caution. Given that both the infectious agent and historical period differ between COVID-19 and prior pandemics, the impact on suicide and related behaviors may also differ.

Given these circumstances, we suggest that a key function of this review is to identify several gaps in the literature that the research community ought to work expeditiously to address (see Table 1). Considering the public health scale of the potential impact of epidemics on suicide-related outcomes, we believe that suicide research and prevention initiatives should be led, commissioned, and funded by local, national, and international governmental bodies, charities, and public health agencies in partnership with research centers and the private sector. Existing research methodologies and approaches might be useful to address the research and prevention gaps, including real-time surveillance strategies (e.g., Cwik et al., 2016), ecological designs on the impact of government support/benefits (e.g., Alves et al., 2019), observational studies on mechanisms and moderating processes (e.g., Brooks et al., 2020), and community outreach and clinical intervention research designed to identify individuals at risk for suicide and to seek to intervene effectively to reduce suicide risk.

**Table 1:** Critical gaps in the literature regarding the impact of infectious disease-related public health emergencies (epidemics) on suicide or suicide-related outcomes

Critical gaps in literature	
1	What is the association between COVID-19 and rates of suicide and related outcomes across regions and cultures both in the short and long term?
2	What is the trajectory of any observed changes (e.g., an initial decrease in suicide outcomes due to a "pulling together" phenomenon followed by a steady increase vs. an initial increase that slowly dissipates)?
3	Are there particular populations (e.g., older adults, frontline healthcare workers, high population density/urban dwellers, men) who are at elevated risk of suicide outcomes during pandemics compared with baseline rates? And, if so, are they amenable to targeted interventions?
4	Are those directly exposed to the virus or their families/caregivers at elevated risk of suicide outcomes, whether immediately or over the longer term?
5	What is the population-attributable risk of suicide outcomes that arises from factors unique to pandemics (e.g., social distancing; mass exposure to a virus with neuropsychiatric health sequelae) versus more general, ongoing risk factors (psychiatric illness, medical illness, access to means)?
6	Which suicide-specific (e.g., media campaigns, means restriction) and nonspecific (social safety net, efforts to reduce social isolation) population-level interventions have the greatest impact on suicide outcomes?
7	Which surveillance strategies are most effective in detecting and intervening to prevent suicide during the pandemic?
8	Can remote or virtual suicide risk assessments be conducted in a sensitive, safe, and effective fashion?
9	If rates of suicide outcomes change, what are the mechanisms or processes (neurobiological, psychological, social) that drive those changes?

## Conclusion 

This comprehensive systematic review constitutes the best available current knowledge regarding the impact of epidemics on suicide-related outcomes. Despite limitations inherent in the study of epidemics (manifested in low methodological quality of available research), the results of the current systematic review suggest a potential association between previous infectious disease-related public health emergencies and increased risk of suicidal thoughts, behavior, and deaths.

## Electronic Supplementary Material 

The electronic supplementary material is available with the online version of the article at https://doi.org/10.1027/0227-5910/a000753

**ESM 1.** Full list of research questions, PRISMA diagrams, full search strategy, inter-rater reliability analysis, data extraction tool, quality assessment framework, additional publications

## Notes

**Conflict of Interest:** TZ reports being employed by the Scottish Government's National Suicide Prevention Leadership Group during the conduct of the study. Outside the submitted work, TZ reports grants from NHS Scotland Endowment Funds and is a co-chair of the Early Career Group of the International Association for Suicide Prevention. MJH reports grants from Canadian Institutes of Health Research, grants from Movember Canada, grants from American Foundation for Suicide Prevention, during the conduct of the study. MS reports grants from American Foundation for Suicide Prevention, grants from Alternative Funding Plan of the Academic Health Sciences Centres of Ontario, grants from University of Toronto Department of Psychiatry Excellence Fund, grants from Ontario Ministry of Research and Innovation, grants from Mental Health Research Canada, grants from Canadian Institutes of Health Research, other from Google Canada, outside the submitted work; NEE reports grants from Canadian Institutes of Health Research, during the conduct of the study. SP reports personal fees from Health Service Executive, Dublin, Ireland, personal fees from NHS Health Scotland, Edinburgh, Scotland, outside the submitted work, and is co-chair of the Academic Advisory Group to the Scottish Government's National Suicide Prevention Leadership Group. RCO'C is a member of the National Institute of Health and Care Excellence's guideline development group for the management of self-harm; is co-chair of the Academic Advisory Group to the Scottish Government's National Suicide Prevention Leadership Group; receives royalties from books, and occasional fees for workshops and invited addresses; and reports grants from Medical Research Foundation, the Mindstep Foundation, Chief Scientist Office, Medical Research Council, NHS Health Scotland, Scottish Government, and National Institute for Health Research (NIHR). All other authors declare no competing interests.

**Authorship:** Author statement following CRediT (Contributor Roles Taxonomy) guidelines (Allen et al., 2019). TZ and CB are joint first authors; MS and SP are joint last authors. Conceptualization: TZ, MJ, HMc, MMT, EA, PC, SH, MJH, PL, RCOC, GG, EW, MS, SP. Methodology: TZ, CB, MJ, HMc, MT, MMT, EA, PC, SH, MJH, PL, RCOC, NEE, GG, EW, MS, SP. Software: TZ. Validation: TZ, CB, MJ, MT, EA, PL, SP. Formal Analysis: TZ, CB, MJ, MJH. Investigation: TZ, CB, MJ, MT, MMT, EA, PC, SH, PL, YC. Resources: TZ, GG. Data Curation: TZ, CB, PC, PL. Writing – Original Draft: TZ, CB, MJ, MJH, MS, SP. Writing – Reviewing and Editing: TZ, CB, MJ, HMc, MT, MMT, EA, PC, SH, MJH, PL, RCOC, NEE, GG, MS, SP. Visualization: TZ, CB, MT, MMT. Supervision: EA, MJH, RCOC, MS, SP. Project Administration: TZ, EA, EW. Funding Acquisition: SH, MJH, NEE, EW, MS. Screening (titles & abstracts and/or full-text): TZ, CB, MJ, HMc, MMT, EA, PC, SH, MJH, PL, RCOC, NEE, YC, GG, MS. The corresponding author attests that all listed authors meet authorship criteria and that no others meeting the criteria have been omitted. TZ and CB act as guarantors.

**Open Data:** A dataset with all abstracts screened by the research team will be made available in the Open Science Framework (https://osf.io/7hzu5/) after the publication of the manuscript.

## Appendix

**Table A1:** Characteristics of included publications

Study, country, and study period	Population	Public Health Emergency	Suicide/Self-harm	Main findings	Quality assessment
Sample/Source	Sex/Age	Exposure/Measures	Main public health responses	Outcomes/Measures
Huang et al. (2005) [Taiwan] Study period: 2003	Emergency department adult patients in a SARS-dedicated hospital.	Adults > 14 years	Exposure: Severe Acute Respiratory Syndrome (SARS). Measures: Not reported.	Not reported.	Attempted suicide via medication self-poisoning. Measures: Emergency Department Medical Records.	Increase in suicide attempts by self-poisoning during peak epidemic stage; not statistically significant.	3
Wasserman (1992) [USA] Study period: 1910–1920	USA citizens. Source: The US Bureau of the Census (1910–1920).	Not reported.	Exposure: The Great Influenza Epidemic//"Spanish Flu." Measures: Spanish Flu: Mortality data from the US Bureau of the Census (1922).	Social distancing (closure of schools, churches, theaters, moving picture halls, dance halls, saloons, and sporting arenas, curtailment of the 1918 political campaign. Some states were forced to don gauze masks).	Outcome: Suicide deaths. Measures: Surveillance data.	Mortality rate during the Spanish Flu (1918–1920) was positively associated with an increase in suicide rates.	5
Honigsbaum (2010) [UK] Study period: 1889–1893	UK citizens (with a focus on Sheffield and other northern towns).	Not reported.	Exposure: Russian influenza. Measure: Historical archives (medical officer of health and national and local newspaper reports, and the poetry and memoirs of prominent survivors).	Not reported.	Outcome: Suicide deaths. Measures: Surveillance data, historical documents.	The epidemic coincided with a marked rise in the suicide rate. Coroners' verdicts of suicide in England and Wales, of whom 60% were male, increased by 25% between 1889 and 1893, and in 1893 the suicide rate peaked at 8.5/100,000, "the highest on record."	5
Yip et al. (2010) [Hong Kong] Study period: 2003	Older adults in Hong Kong aged > 65 who died by suicide that was SARS-related. *N* = 22. Source: Coroner Court reports.	Sex: M = 11, F = 11. Mean age: 74.9 (≥ 65), general population.	Exposure: Severe Acute Respiratory Syndrome (SARS). Measure: Number of deaths from confirmed affected individuals.	Quarantine actions at several hospitals and hotspots to control the spread of the disease. In addition, social contact and networking within the community was reduced to minimize the epidemic's spread.	Outcome: Suicide deaths that were SARS-related. Measures: Suicide notes and witnesses' descriptions of the suicide deaths.	SARS-related older-adult suicide dead were more likely to be afraid of contracting the disease (χ^2^ = 29.33, *df* = 1, *p* < .001) and had fears of disconnection (χ^2^ = 9.26, *df* = 1, *p* < .002). The SARS-related suicide dead feared being a burden to their families during the epidemic. No significant differences in sociodemographics, employment status, medical or psychiatric profiles, and level of dependence on others.	4
Keita et al. (2017) [Guinea, West Africa]Study period: 23/03/15–11/07/16.	*N* = 256 for total study. *n* = 33 for clinical observation with a psychiatrist. Individuals aged ≥ 20, participating in the PostEboGui study, who were receiving care at the Conakry site, and who had completed the CES-D.	Sex: M = 118; F = 138. Age median: 32 (26–41).	Exposure: Ebola virus disease (EVD). Measure: Having EVD confirmed by laboratory exams and being admitted to the Ebola Treatment Center for treatment.	Not reported.	Outcomes: Suicidal ideation and suicide attempt. Measures: Clinical interview with a psychiatrist.	Thirty-eight participants (15%) had a score higher than the threshold value of the CES-D for depressive symptoms. In 33 participants who had a clinical consultation with a psychiatrist following completion of the CES-D, 1 person presented with suicidal ideation and 3 participants had attempted suicide.	3
Chan et al. (2006) [Hong Kong] Study period: 1986–2003.	All individuals aged ≥ 65 who died by suicide in Hong Kong during 1986–2003. Source: Census & Statistics Department of the Government of Hong Kong Special Administrative Region.	Sex: Not reported. Age: ≥ 65.	Exposure: Severe Acute Respiratory Syndrome (SARS). Measure: Number of deaths from confirmed affected individuals.	Resources were channeled to combating SARS at the expense of routine nonemergency healthcare services. Widespread disruptions in social networking were evident as most residents in Hong Kong minimized their outings.	Outcome: Suicide deaths. Measures: Surveillance data.	There was a significant rise in older adult suicide rates from 2002 to 2003 (IRR = 1.32, *p* = .002). This increase reached statistical significance for women (IRR = 1.42; *p* = .014) but not for men (IRR = 1.22; *p* = .087) or those under 65 (IRR = 0.97; *p* = .48).	5
Cheung et al. (2008) [Hong Kong] Study period: 1993–2004.	All individuals ≥ 65 years of age who died by suicide in Hong Kong during 1993–2004. *N* = 321 (detailed information obtained for *n* = 303). Source: Hong Kong Coroners' Court.	Sex: M = 181; F = 122. Age: ≥ 65.	Exposure: Severe Acute Respiratory Syndrome (SARS). Measure: Number of deaths from confirmed affected individuals.	Due to the fear of contracting SARS, older adults reduced social contacts and were housebound voluntarily and/or involuntarily. Besides, the quarantine measures imposed to curtail the spread of the epidemic also played a role in weakening social networks.	Outcome: Suicide deaths. Measures: Surveillance data.	Results showed an excess of older adult suicides in April 2003, when compared with April of previous years. The annual older-adult suicide rates in 2003 and 2004 were significantly higher than that in 2002, suggesting the suicide rate did not return to the level before the SARS epidemic. Overall severity of illness (χ^2^ = 25.104, *df* = 6, *p* < .001), level of dependency (χ^2^ = 12.697, *df* = 6, *p* < .013), and worrying about having sickness (χ^2^ = 7.721, *df* = 2, *p* < .021) among the older adult suicides were found to be significantly different in the pre-, peri-, and post-SARS periods.	6
Okusaga et al. (2011) [USA] Study period: not provided.	Clinical sample of mood disorder patients versus healthy controls.	Sex (depressed sample): M = 95; F = 162. Mean age: 43.4 (*SD* = 10.9).	Seropositivity for coronaviruses, influenza A and B viruses; not related to particular epidemic exposure.	Not applicable.	Columbia Suicide History Form Interview	Among individuals with a history of mood disorder, seropositivity for influenza B was significantly associated with a history of suicide attempt(s), 96 (97.0%) versus 104 (83.9%; *p* = .001), and the odds of having attempted suicide were increased in influenza B seropositive individuals (*OR* = 2.53, 95% CI [1.33, 4.80]). No association with influence A or coronaviruses.	1
