# Supplementary material for: The Impact of Infectious Disease-Related Public Health Emergencies on Suicide, Suicidal Behavior, and Suicidal Thoughts: A Systematic Review
Source: Crisis. 2020 Oct 16;42(6):474–87. doi: 10.1027/0227-5910/a000753 (PMC8689932; doi:10.1027/0227-5910/a000753)
Supplement: Supplementary file 1 [file cri_42_6_474_esm1.pdf]

Electronic Supplementary Material

Contents

Full List of Research Questions 2
PRISMA Diagrams 3
Figure A. PRISMA Diagram for Search 1 (S1) and Search 2 (S2) 3
Figure B. PRISMA Diagram for Search 4 (S4) 4
Full Search Strategy 5
Searches 5
Main search 5
Grey literature search 11
Inter-rater Reliability Analysis 12
Data Extraction Tool 13
Quality Assessment Framework 14
Additional Publications on the COVID-19 Pandemic and Suicide-Related Outcomes 15
Preprint 15
COVID-19 Case Studies 16
References 17

Full List of Research Questions

The research questions guiding this systematic review include: 1) Does exposure to an infectious disease-related public health emergency result in different levels of risk: (a) across age groups (adolescents and young adults; adults; older adults), (b) by sex and gender, (c) across different socio-demographic and economic groups, (d) in people who have been infected compared to those who have not, and (e) in those with pre-existing health conditions (including mental health disorders); (f) does the literature report other groups with increased risk of suicide after an infectious disease-related public health emergency? 2) What can we learn from previous infectious disease-related public health emergencies about the likely impact of the COVID-19 pandemic on the incidence of suicidal behavior, suicidal thoughts, and self-harm? 3) What can we learn from previous infectious disease-related public health emergencies about the likely effectiveness of different interventions to mitigate the impact of the COVID-19 pandemic on the incidence of suicidal behavior, suicidal thoughts, and self-harm?

PRISMA Diagrams

Figure A. PRISMA Diagram for Search 1 (S1) and Search 2 (S2)

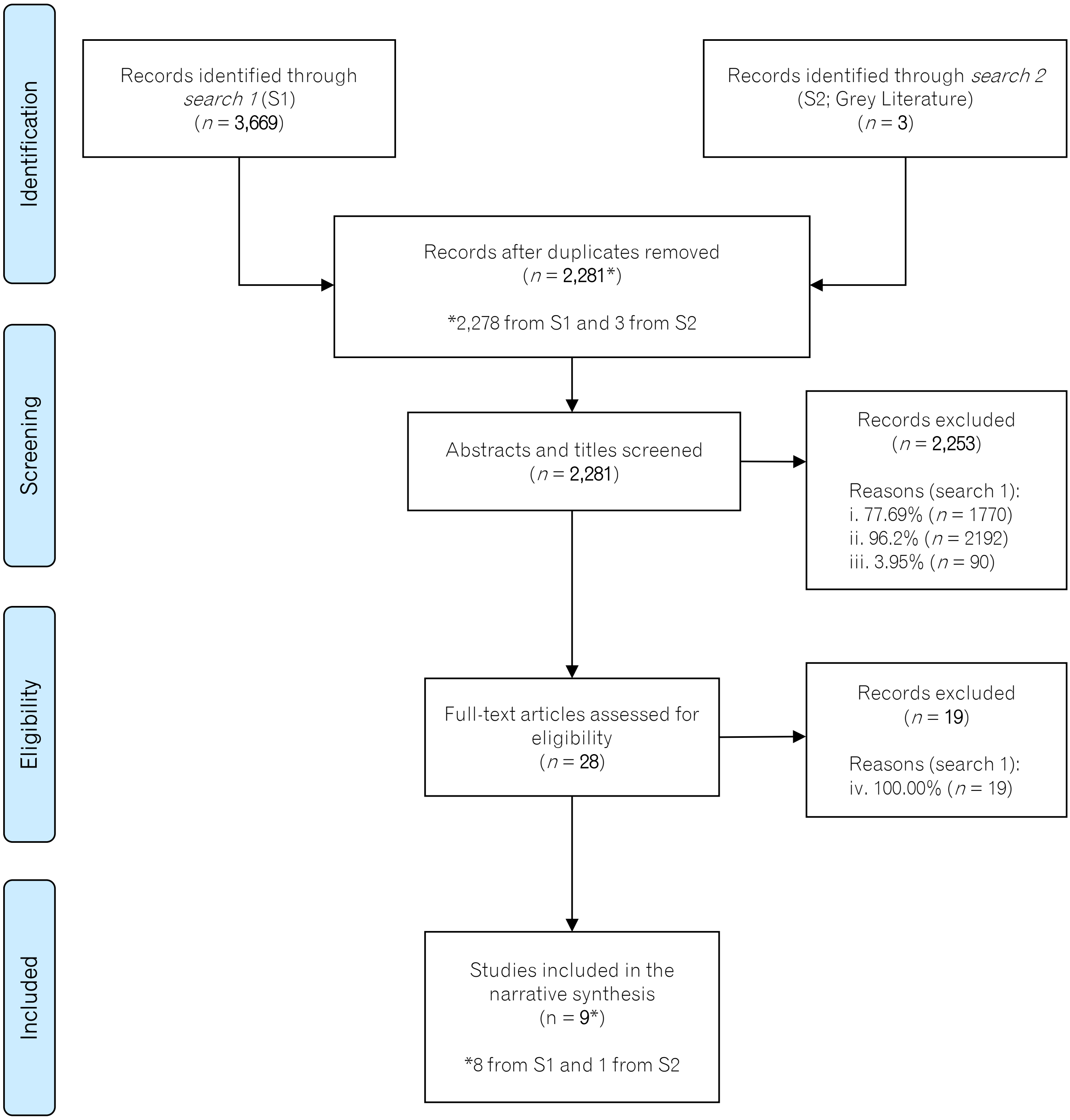

Search 1 and 2 (S1 and S2) PRISMA Diagram: Preferred reporting items for systematic reviews and meta-analyses (PRISMA) flow diagram. Reasons for exclusion: (i) the publication did not focus on suicidal behaviour, suicidal ideation and/or self-harm; (ii) the publication did not focus on any infectious disease-related public health emergency; (iii) The publication was excluded due to its publication type (not a primary study, systematic review, or meta-analysis); (iv) The publication did not contain empirical data eligible for data extraction and quality assessment; and (v) the publication was a duplicate of another in the study pool, missed in the initial duplicate removal.

Figure B. PRISMA Diagram for Search 4 (S4)

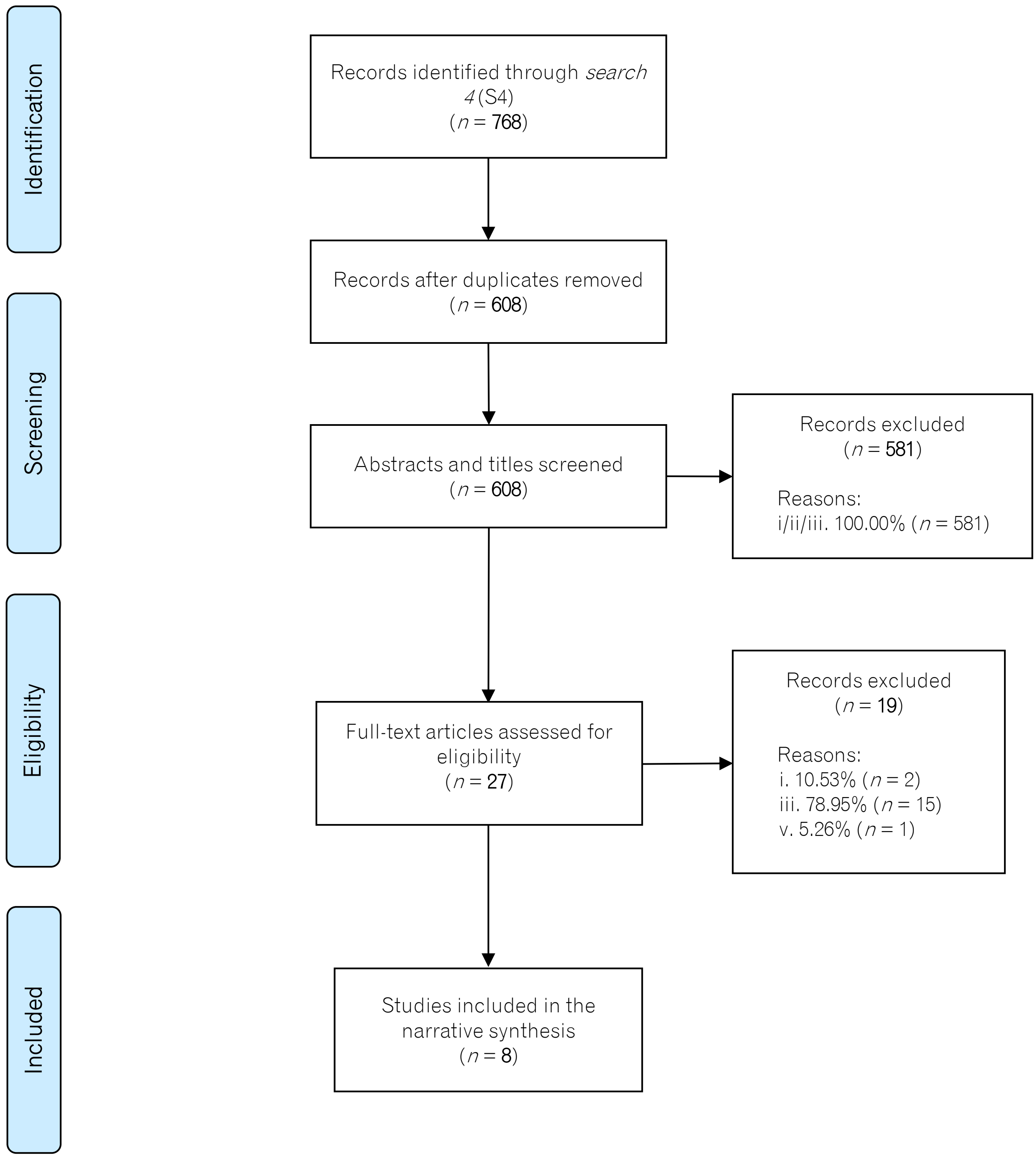

Search 4 (S4) PRISMA Diagram: Preferred reporting items for systematic reviews and meta-analyses (PRISMA) flow diagram. Reasons for exclusion: (i) the publication did not focus on suicidal behaviour, suicidal ideation and/or self-harm; (ii) the publication did not focus on any infectious disease-related public health emergency; (iii) The publication was excluded due to its publication type (not a primary study, systematic review, or meta-analysis); (iv) The publication did not contain empirical data eligible for data extraction and quality assessment; and (v) the publication was a duplicate of another in the study pool, missed in the initial duplicate removal.

Full Search Strategy

Searches

Main search

MEDLINE and EMBASE were searched using keywords and the appropriate subject headings:
MEDLINE (Ovid) Search History

1	exp Suicide/ or suicide.mp.	85129	Advanced
2	suicides.mp. [mp=title, abstract, original title, name of substance word, subject heading word, floating sub-heading word, keyword heading word, organism supplementary concept word, protocol supplementary concept word, rare disease supplementary concept word, unique identifier, synonyms]	7879	Advanced
3	exp Self-Injurious Behavior/ or self-harm.mp.	72295	Advanced
4	("self-injurious behavior" or "self injurious behavior" or "self-injurious behaviour" or "self injurious behaviour").mp. [mp=title, abstract, original title, name of substance word, subject heading word, floating sub-heading word, keyword heading word, organism supplementary concept word, protocol supplementary concept word, rare disease supplementary concept word, unique identifier, synonyms]	8570	Advanced
5	"self harm".mp. [mp=title, abstract, original title, name of substance word, subject heading word, floating sub-heading word, keyword heading word, organism supplementary concept word, protocol supplementary concept word, rare disease supplementary concept word, unique identifier, synonyms]	5597	Advanced
6	self-injury.mp.	2928	Advanced
7	"self injury".mp. [mp=title, abstract, original title, name of substance word, subject heading word, floating sub-heading word, keyword heading word, organism supplementary concept word, protocol supplementary concept word, rare disease supplementary concept word, unique identifier, synonyms]	2928	Advanced
8	suicidal ideation.mp. or exp Suicidal Ideation/	12947	Advanced
9	self-poisoning.mp.	1705	Advanced
10	"self poisoning".mp. [mp=title, abstract, original title, name of substance word, subject heading word, floating sub-heading word, keyword heading word, organism supplementary concept word, protocol supplementary concept word, rare disease supplementary concept word, unique identifier, synonyms]	1705	Advanced
11	suicidal poisoning.mp.	176	Advanced
12	suicidal.mp.	29469	Advanced
13	("suicidal overdose" or "intentional overdose" or "intentional drug overdose").mp. [mp=title, abstract, original title, name of substance word, subject heading word, floating sub-heading word, keyword heading word, organism supplementary concept word, protocol supplementary concept word, rare disease supplementary concept word, unique identifier, synonyms]	326	Advanced
14	automutilation.mp.	102	Advanced

15	auto-mutilation.mp. [mp=title, abstract, original title, name of substance word, subject heading word, floating sub-heading word, keyword heading word, organism supplementary concept word, protocol supplementary concept word, rare disease supplementary concept word, unique identifier, synonyms]	21	Advanced
16	self mutilation.mp. or exp Self Mutilation/	3823	Advanced
17	self-mutilation.mp. [mp=title, abstract, original title, name of substance word, subject heading word, floating sub-heading word, keyword heading word, organism supplementary concept word, protocol supplementary concept word, rare disease supplementary concept word, unique identifier, synonyms]	3823	Advanced
18	parasuicide.mp. [mp=title, abstract, original title, name of substance word, subject heading word, floating sub-heading word, keyword heading word, organism supplementary concept word, protocol supplementary concept word, rare disease supplementary concept word, unique identifier, synonyms]	486	Advanced
19	suicidal thoughts.mp.	2583	Advanced
20	suicidal thought.mp. [mp=title, abstract, original title, name of substance word, subject heading word, floating sub-heading word, keyword heading word, organism supplementary concept word, protocol supplementary concept word, rare disease supplementary concept word, unique identifier, synonyms]	85	Advanced
21	self-cutting.mp. [mp=title, abstract, original title, name of substance word, subject heading word, floating sub-heading word, keyword heading word, organism supplementary concept word, protocol supplementary concept word, rare disease supplementary concept word, unique identifier, synonyms]	182	Advanced
22	"self cutting".mp. [mp=title, abstract, original title, name of substance word, subject heading word, floating sub-heading word, keyword heading word, organism supplementary concept word, protocol supplementary concept word, rare disease supplementary concept word, unique identifier, synonyms]	182	Advanced
23	1 or 2 or 3 or 4 or 5 or 6 or 7 or 8 or 9 or 10 or 11 or 12 or 13 or 14 or 15 or 16 or 17 or 18 or 19 or 20 or 21 or 22	103016	Advanced
24	pandemic.mp. or Pandemics/	29306	Advanced
25	pandemics.mp. [mp=title, abstract, original title, name of substance word, subject heading word, floating sub-heading word, keyword heading word, organism supplementary concept word, protocol supplementary concept word, rare disease supplementary concept word, unique identifier, synonyms]	10064	Advanced
26	epidemic.mp. or exp Epidemics/	99581	Advanced
27	epidemics.mp. [mp=title, abstract, original title, name of substance word, subject heading word, floating sub-heading word, keyword heading word, organism supplementary concept word, protocol supplementary concept word, rare disease supplementary concept word, unique identifier, synonyms]	29897	Advanced
28	outbreaks.mp. or exp Disease Outbreaks/	124672	Advanced
29	outbreak.mp. [mp=title, abstract, original title, name of substance word, subject heading word, floating sub-heading word, keyword heading word, organism supplementary concept word, protocol supplementary concept word, rare disease supplementary concept word, unique identifier, synonyms]	58600	Advanced
30	public health emergency.mp.	1647	Advanced
31	"public health emergencies".mp. [mp=title, abstract, original title, name of substance word, subject heading word, floating sub-heading word, keyword heading word, organism supplementary concept word, protocol supplementary concept word, rare disease supplementary concept word, unique identifier, synonyms]	785	Advanced
32	24 or 25 or 26 or 27 or 28 or 29 or 30 or 31	232905	Advanced
33	influenza.mp. or exp Influenza, Human/	110126	Advanced
34	Influenza A Virus.mp. or exp Influenza A virus/	46436	Advanced
35	influenza pandemic.mp.	3352	Advanced
36	H2N2.mp.	672	Advanced
37	H3N2.mp. [mp=title, abstract, original title, name of substance word, subject heading word, floating sub-heading word, keyword heading word, organism supplementary concept word, protocol supplementary concept word, rare disease supplementary concept word, unique identifier, synonyms]	7721	Advanced
38	exp Influenza Pandemic, 1918-1919/ or Spanish flu.mp.	307	Advanced
39	Asian flu.mp.	44	Advanced
40	Hong Kong flu.mp.	30	Advanced
41	exp Ebolavirus/ or exp Hemorrhagic Fever, Ebola/ or Ebola.mp.	9132	Advanced
42	exp SARS Virus/ or SARS.mp. or exp Severe Acute Respiratory Syndrome/	13542	Advanced
43	"Severe acute respiratory syndrome".mp. [mp=title, abstract, original title, name of substance word, subject heading word, floating sub-heading word, keyword heading word, organism supplementary concept word, protocol supplementary concept word, rare disease supplementary concept word, unique identifier, synonyms]	10036	Advanced
44	exp Coronavirus Infections/ or exp Coronavirus/ or Coronavirus.mp.	24815	Advanced
45	MERS.mp. or exp Middle East Respiratory Syndrome Coronavirus/	4675	Advanced

	46	"Middle East Respiratory Syndrome".mp. [mp=title, abstract, original title, name of substance word, subject heading word, floating sub-heading word, keyword heading word, organism supplementary concept word, protocol supplementary concept word, rare disease supplementary concept word, unique identifier, synonyms]	2206	Advanced
	47	Covid.mp. or exp Betacoronavirus/	17705	Advanced
	48	Betacoronavirus.mp. [mp=title, abstract, original title, name of substance word, subject heading word, floating sub-heading word, keyword heading word, organism supplementary concept word, protocol supplementary concept word, rare disease supplementary concept word, unique identifier, synonyms]	2569	Advanced
	49	covid-19.mp. [mp=title, abstract, original title, name of substance word, subject heading word, floating sub-heading word, keyword heading word, organism supplementary concept word, protocol supplementary concept word, rare disease supplementary concept word, unique identifier, synonyms]	11328	Advanced
	50	Swine flu.mp.	956	Advanced
	51	exp Zika Virus Infection/ or exp Zika Virus/ or zika.mp.	7790	Advanced
	52	Arbovirus.mp. or exp Arboviruses/	9334	Advanced
	53	Arboviruses.mp. [mp=title, abstract, original title, name of substance word, subject heading word, floating sub-heading word, keyword heading word, organism supplementary concept word, protocol supplementary concept word, rare disease supplementary concept word, unique identifier, synonyms]	5816	Advanced
	54	Dengue.mp. or exp Dengue/ or exp Severe Dengue/ or exp Dengue Virus/	22867	Advanced
	55	Yellow Fever/ or Yellow fever.mp.	6871	Advanced
	56	Hemorrhagic fever.mp.	13929	Advanced
	57	Haemorrhagic fever.mp.	2098	Advanced
	58	exp Encephalitis/ or Encephalitis.mp.	66978	Advanced
	59	exp Plague/ or Plague.mp.	10458	Advanced
	60	Bubonic plague.mp.	555	Advanced
	61	exp Smallpox/ or Smallpox.mp.	9866	Advanced
	62	"small pox".mp. [mp=title, abstract, original title, name of substance word, subject heading word, floating sub-heading word, keyword heading word, organism supplementary concept word, protocol supplementary concept word, rare disease supplementary concept word, unique identifier, synonyms]	735	Advanced
	63	H1N1.mp. [mp=title, abstract, original title, name of substance word, subject heading word, floating sub-heading word, keyword heading word, organism supplementary concept word, protocol supplementary concept word, rare disease supplementary concept word, unique identifier, synonyms]	20940	Advanced
	64	H7N9.mp. [mp=title, abstract, original title, name of substance word, subject heading word, floating sub-heading word, keyword heading word, organism supplementary concept word, protocol supplementary concept word, rare disease supplementary concept word, unique identifier, synonyms]	1920	Advanced
	65	SARS-COV-3.mp. [mp=title, abstract, original title, name of substance word, subject heading word, floating sub-heading word, keyword heading word, organism supplementary concept word, protocol supplementary concept word, rare disease supplementary concept word, unique identifier, synonyms]	2	Advanced
	66	exp Chikungunya virus/ or exp Chikungunya Fever/ or Chikungunya.mp.	5489	Advanced
	67	Cholera.mp. or exp Cholera/	27528	Advanced
	68	("Crimean-Congo haemorrhagic fever" or "Crimean Congo haemorrhagic fever" or "Crimean-Congo hemorrhagic fever" or "Crimean Congo hemorrhagic fever").mp. [mp=title, abstract, original title, name of substance word, subject heading word, floating sub-heading word, keyword heading word, organism supplementary concept word, protocol supplementary concept word, rare disease supplementary concept word, unique identifier, synonyms]	1394	Advanced
	69	Hendra virus.mp. or exp Hendra Virus/	473	Advanced
	70	Lassa fever.mp. or exp Lassa Fever/	1029	Advanced
	71	exp Marburgvirus/ or exp Marburg Virus Disease/ or Marburg.mp.	2356	Advanced
	72	Meningitis.mp. or exp Meningitis/	75429	Advanced
	73	MERS-CoV.mp. [mp=title, abstract, original title, name of substance word, subject heading word, floating sub-heading word, keyword heading word, organism supplementary concept word, protocol supplementary concept word, rare disease supplementary concept word, unique identifier, synonyms]	1766	Advanced
	74	exp Monkeypox virus/ or exp Monkeypox/ or Monkeypox.mp.	801	Advanced
	75	Nipah virus.mp. or exp Nipah Virus/	892	Advanced
	76	Rift valley fever.mp. or exp Rift Valley Fever/	1974	Advanced
	77	exp Tularemia/ or Tularaemia.mp.	3486	Advanced

78	Tularemia.mp. [mp=title, abstract, original title, name of substance word, subject heading word, floating sub-heading word, keyword heading word, organism supplementary concept word, protocol supplementary concept word, rare disease supplementary concept word, unique identifier, synonyms]	4114	Advanced
79	33 or 34 or 35 or 36 or 37 or 38 or 39 or 40 or 41 or 42 or 43 or 44 or 45 or 46 or 47 or 48 or 49 or 50 or 51 or 52 or 53 or 54 or 55 or 56 or 57 or 58 or 59 or 60 or 61 or 62 or 63 or 64 or 65 or 66 or 67 or 68 or 69 or 70 or 71 or 72 or 73 or 74 or 75 or 76 or 77 or 78	376006	Advanced
80	23 and 32 and 79	59	Advanced
81	23 and 79	362	Advanced
82	80 or 81	362	Advanced

EMBASE (Ovid) Search History

1	exp suicide/ or Suicide.mp.	112332	Advanced
2	"suicides".mp. [mp=title, abstract, heading word, drug trade name, original title, device manufacturer, drug manufacturer, device trade name, keyword, floating subheading word, candidate term word]	10398	Advanced
3	self-harm.mp. or exp automutilation/	21146	Advanced
4	Self-injurious behaviour.mp.	567	Advanced
5	self-injury.mp.	3649	Advanced
6	("self harm" or "self injurious behaviour" or "self-injurious behavior" or "self injurious behavior").mp. [mp=title, abstract, heading word, drug trade name, original title, device manufacturer, drug manufacturer, device trade name, keyword, floating subheading word, candidate term word]	9792	Advanced
7	Suicidal Ideation.mp. or exp suicidal ideation/	23700	Advanced
8	self-poisoning.mp. or exp self poisoning/	3038	Advanced
9	("self poisoning" or "suicidal poisoning").mp. [mp=title, abstract, heading word, drug trade name, original title, device manufacturer, drug manufacturer, device trade name, keyword, floating subheading word, candidate term word]	3318	Advanced
10	"suicidal".mp. [mp=title, abstract, heading word, drug trade name, original title, device manufacturer, drug manufacturer, device trade name, keyword, floating subheading word, candidate term word]	52319	Advanced
11	("suicidal overdose" or "intentional overdose" or "intentional drug overdose").mp. [mp=title, abstract, heading word, drug trade name, original title, device manufacturer, drug manufacturer, device trade name, keyword, floating subheading word, candidate term word]	537	Advanced
12	("auto mutilation" or "auto-mutilation" or "self mutilation" or "self-mutilation").mp. [mp=title, abstract, heading word, drug trade name, original title, device manufacturer, drug manufacturer, device trade name, keyword, floating subheading word, candidate term word]	2076	Advanced
13	parasuicide.mp.	678	Advanced
14	("suicidal thoughts" or "suicidal thought").mp. [mp=title, abstract, heading word, drug trade name, original title, device manufacturer, drug manufacturer, device trade name, keyword, floating subheading word, candidate term word]	3462	Advanced
15	self-cutting.mp.	244	Advanced
16	self cutting.mp.	244	Advanced
17	1 or 2 or 3 or 4 or 5 or 6 or 7 or 8 or 9 or 10 or 11 or 12 or 13 or 14 or 15 or 16	148859	Advanced
18	exp pandemic influenza/ or exp pandemic/ or pandemic.mp.	33973	Advanced
19	"pandemics".mp. [mp=title, abstract, heading word, drug trade name, original title, device manufacturer, drug manufacturer, device trade name, keyword, floating subheading word, candidate term word]	4036	Advanced
20	epidemic.mp. or exp epidemic/	186709	Advanced
21	"epidemics".mp. [mp=title, abstract, heading word, drug trade name, original title, device manufacturer, drug manufacturer, device trade name, keyword, floating subheading word, candidate term word]	26367	Advanced
22	outbreak.mp.	68760	Advanced
23	"outbreaks".mp. [mp=title, abstract, heading word, drug trade name, original title, device manufacturer, drug manufacturer, device trade name, keyword, floating subheading word, candidate term word]	54783	Advanced
24	public health emergency.mp.	1748	Advanced
25	"public health emergencies".mp. [mp=title, abstract, heading word, drug trade name, original title, device manufacturer, drug manufacturer, device trade name, keyword, floating subheading word, candidate term word]	775	Advanced
26	18 or 19 or 20 or 21 or 22 or 23 or 24 or 25	282275	Advanced
27	exp influenza/ or Influenza.mp. or exp influenza A/	163099	Advanced
28	"influenza pandemic".mp. [mp=title, abstract, heading word, drug trade name, original title, device manufacturer, drug manufacturer, device trade name, keyword, floating subheading word, candidate term word]	3862	Advanced

29	H2N2.mp. or exp "Influenza A virus (H2N2)"/	1695	Advanced
30	H3N2.mp. or exp "Influenza A virus (H3N2)"/	10509	Advanced
31	spanish flu.mp. or exp Spanish influenza/	654	Advanced
32	"spanish influenza".mp. [mp=title, abstract, heading word, drug trade name, original title, device manufacturer, drug manufacturer, device trade name, keyword, floating subheading word, candidate term word]	655	Advanced
33	asian flu.mp. or exp Asian influenza/	443	Advanced
34	hong kong flu.mp. or exp Hong Kong influenza/	263	Advanced
35	("asian influenza" or "hong kong influenza").mp. [mp=title, abstract, heading word, drug trade name, original title, device manufacturer, drug manufacturer, device trade name, keyword, floating subheading word, candidate term word]	957	Advanced
36	ebola.mp. or exp Ebola hemorrhagic fever/	11671	Advanced
37	SARS.mp. or exp severe acute respiratory syndrome/	16466	Advanced
38	"severe acute respiratory syndrome".mp. [mp=title, abstract, heading word, drug trade name, original title, device manufacturer, drug manufacturer, device trade name, keyword, floating subheading word, candidate term word]	11795	Advanced
39	Covid.mp.	7105	Advanced
40	coronavirus.mp. or exp Coronavirinae/	26244	Advanced
41	"Covid-19".mp. [mp=title, abstract, heading word, drug trade name, original title, device manufacturer, drug manufacturer, device trade name, keyword, floating subheading word, candidate term word]	7050	Advanced
42	MERS.mp.	4894	Advanced
43	middle east respiratory syndrome.mp. or exp Middle East respiratory syndrome/	3006	Advanced
44	swine flu.mp. or exp swine influenza/	1984	Advanced
45	"swine influenza".mp. [mp=title, abstract, heading word, drug trade name, original title, device manufacturer, drug	3108	Advanced
46	exp Zika virus/ or zika.mp. or exp Zika fever/	10344	Advanced
47	Arbovirus.mp. or exp Arbovirus/	9399	Advanced
48	Dengue.mp. or exp dengue/ or exp Dengue virus/ or exp severe dengue/	32632	Advanced
49	yellow fever.mp. or exp yellow fever/	10142	Advanced
50	hemorrhagic fever.mp. or exp hemorrhagic fever/	25962	Advanced
51	"haemorrhagic fever".mp. [mp=title, abstract, heading word, drug trade name, original title, device manufacturer, drug manufacturer, device trade name, keyword, floating subheading word, candidate term word]	2864	Advanced
52	encephalitis.mp. or exp encephalitis/	136154	Advanced
53	exp plague/ or plague.mp.	13519	Advanced
54	"bubonic plague".mp. [mp=title, abstract, heading word, drug trade name, original title, device manufacturer, drug manufacturer, device trade name, keyword, floating subheading word, candidate term word]	646	Advanced
55	smallpox.mp. or exp smallpox/ or exp Smallpox virus/	14934	Advanced
56	"small pox".mp. [mp=title, abstract, heading word, drug trade name, original title, device manufacturer, drug manufacturer, device trade name, keyword, floating subheading word, candidate term word]	400	Advanced
57	("H1N1" or "H7N9" or "SARS-COV-3").mp. [mp=title, abstract, heading word, drug trade name, original title, device manufacturer, drug manufacturer, device trade name, keyword, floating subheading word, candidate term word]	30803	Advanced
58	chikungunya.mp. or exp Chikungunya virus/ or exp chikungunya/	8143	Advanced
59	exp cholera/ or cholera.mp.	33314	Advanced
60	Hendra virus.mp. or exp Hendra virus/	677	Advanced
61	Lassa Fever.mp. or exp Lassa fever/	1289	Advanced
62	exp Marburg hemorrhagic fever/ or Marburg.mp. or exp Marburg virus/	3723	Advanced

☐	63	exp meningitis/ or meningitis.mp.	122368	Advanced
☐	64	"MERS-CoV".mp. [mp=title, abstract, heading word, drug trade name, original title, device manufacturer, drug manufacturer, device trade name, keyword, floating subheading word, candidate term word]	1831	Advanced
☐	65	Monkeypox.mp. or exp monkeypox/ or exp Monkeypox virus/	1148	Advanced
☐	66	Nipah Virus.mp. or exp Nipah virus/	1314	Advanced
☐	67	Rift Valley Fever.mp. or exp Rift Valley fever/	2498	Advanced
☐	68	Tularemia.mp. or exp tularemia/	5187	Advanced
☐	69	"tularaemia".mp. [mp=title, abstract, heading word, drug trade name, original title, device manufacturer, drug manufacturer, device trade name, keyword, floating subheading word, candidate term word]	846	Advanced
☐	70	27 or 28 or 29 or 30 or 31 or 32 or 33 or 34 or 35 or 36 or 37 or 38 or 39 or 40 or 41 or 42 or 43 or 44 or 45 or 46 or 47 or 48 or 49 or 50 or 51 or 52 or 53 or 54 or 55 or 56 or 57 or 58 or 59 or 60 or 61 or 62 or 63 or 64 or 65 or 66 or 67 or 68 or 69	552000	Advanced
☐	71	17 and 26 and 70	122	Advanced
☐	72	17 and 70	1393	Advanced
☐	73	71 or 72	1393	Advanced

A keyword search was conducted in PsycInfo, PsycArticles, CINAHL, Scopus, and Web of Science (including Web of Science Core Collection, BIOSIS Citation Index, BIOSIS Previews, CABI: CAB Abstracts, Current Contents Connect, Data Citation Index, Derwent Innovations Index, KCI-Korean Journal Database, Russian Science Citation Index, SciELO Citation Index, Zoological Record) using the following search strategy:

- Concept #1: Suicide.

Suicide OR suicides OR self-harm OR “self harm” OR “self injury” OR self-injury OR “self-injurious behavior” OR “self-injurious behaviour” OR “self injurious behavior” OR “self injurious behaviour” OR “self cutting” OR self-cutting OR “suicide ideation” OR “suicidal ideation” OR “self poisoning” OR self-poisoning OR “suicidal poisoning” OR suicidal OR “suicidal overdose” OR “intentional drug overdose” OR “intentional overdose” OR automutilation OR auto-mutilation OR “self mutilation” OR self-mutilation OR parasuicide OR “suicidal thought” OR “suicidal thoughts”

- Concept #2: Pandemic.

Pandemic OR Pandemics OR Epidemic OR Epidemics OR Outbreak OR Outbreaks OR “Disease outbreaks” OR “Public health emergency” OR “Public health emergencies”

- Concept #3: Viruses and other infectious diseases.

Influenza OR “influenza A virus” OR “influenza pandemic” OR h2n2 OR h3n2 OR “Spanish flu” OR “Asian flu” OR “hong kong flu” OR ebola OR sars OR “severe acute respiratory syndrome” OR coronavirus OR “coronavirus infection” OR mers OR “middle east respiratory syndrome” OR covid OR betacoronavirus OR covid-19 OR “swine flu” OR zika OR arbovirus OR arboviruses OR dengue OR “yellow fever” OR “hemorrhagic fever” OR “haemorrhagic fever” OR encephalitis OR plague OR “sars virus” OR “zika virus” OR “zika virus infection” OR “bubonic plague” OR smallpox OR “small pox” OR h1n1 OR h7n9 OR chikungunya OR cholera OR “Crimean-congo hemorrhagic fever” OR “Crimean-congo haemorrhagic fever” OR “Crimean congo hemorrhagic fever” OR “Crimean congo haemorrhagic fever” OR “Hendra virus” OR “lassa fever” OR Marburg OR meningitis OR mers-cov OR monkeypox OR “nipah virus” OR “rift valley fever” OR tularaemia OR tularemia

Strategy = (Concept #1 AND Concept #2 AND Concept #3) OR (Concept #1 AND Concept #3).

Grey literature search

A complementary search of preprints on the current pandemic was conducted in *PsyArXiv*, *medRxiv*, and *bioRxiv*.

- Concept #1: Suicide.

Suicide OR Suicides OR Self-harm OR Self-injury OR “Self-injurious behaviour” OR “Self-injurious behavior” OR “Suicidal ideation” OR Self-poisoning OR “Self poisoning” OR “Suicidal poisoning” OR automutilation OR “suicidal overdose” OR “Self harm” OR “Self injury” OR “Self mutilation” OR Self-mutilation OR Parasuicide OR “Intentional drug overdose” OR “Suicidal thoughts” OR “Suicidal thought” OR Self-cutting OR “Self cutting”

- Concept #2: COVID-19.

covid-19 OR coronavirus OR 2019-ncov OR sars-cov-2 OR cov-19

Strategy = (Concept #1 AND Concept #2)

Inter-rater Reliability Analysis

Table A. Cohen's kappa estimates

Stage 1 - Titles and abstracts screening							
Pair	*n* Abstracts	*Z*	*p*	*Cohen's kappa*	95% CI	% agree	Judgement
1	242	0.88	0.187	0.39	–0.28; 1.07	98.7	Fair
2	229	4.30	< 0.0001	0.55	0.37; 0.73	90.8	Moderate
3	223	3.25	< 0.0001	0.64	0.40; 0.88	96.4	Substantial
Stage 2 - Full text screening							
Pair	*n* Papers	*Z*	*p*	*Cohen's kappa*	95% CI	% agree	Judgement
4	10	–	–	–	–	100	Full agreement
5	10	–	–	–	–	100	Full agreement
6	9	1.15	0.124	0.61	–0.09; 1.33	90	Substantial

Note: Pairs: 1 = CB & MMT; 2 = NEE & HMc; 3 = MJ & PC; 4 = EA & ROC; 5 = NEE & PL; 6 = SH & YC. Judgement: The judgement for the estimated kappa about the extent of agreement (Landis & Koch, 1977): If *kappa* is less than 0, "No agreement", if 0–0.2, "Slight agreement", if 0.2–0.4, "Fair agreement", if 0.4–0.6, "Moderate agreement", if 0.6–0.8, "Substantial agreement", if 0.8–1.0, "Almost perfect agreement".

All disagreements at stage 1 were resolved through discussions between pairs. A third referee (CB) assessed the eligibility of one publication that remained in disagreement for pair 6 (Falster et al., 2009), and decided upon its exclusion: the paper is outside of the scope of infectious disease-related public health emergencies, and suicide is not a primary condition.

Data Extraction Tool

Table B. Data extraction tool

Title:	
Authors:	
Journal:	Year:
Keywords:	
Aims:	
Study Design:	

Sample:

N:	Mean age & age range:	Country:	Gender: M = F = Other =
Sample:			
Inclusion criteria:			
Exclusion criteria:			
Ethnicity:			
Sexuality:			
Study period (not the publication date):			

Measures:

Suicidal thoughts and behaviors (including fatal Suicide attempt)

What was measured? (Constructs): (1) Suicide; (2) Suicidal thoughts/ideation; (3) Non-fatal self-harm (including suicide attempts).
Instrument: (1) Self-administered; (2) Experimenter/Interviewer Administered; (3) Surveillance/routine data?:

Self-harm (including fatal Self-harm)

What was measured? (Constructs): (1) Self-harm; (2) Self-harm thoughts/ideation
Instrument: (1) Self-administered or (2) Experimenter/ Administered?:

Public Health Emergency

What was it?
How was it assessed?
Main public health responses:

Results:

Main findings:
Effect sizes (specify type of effect size):
Potential mechanisms/Pathways to suicide/self-harm/other mental health co-morbidities:
Author's Interpretations:
Author's Limitations:

Reviewer's comments:

Quality Assessment Framework

Table C. Quality assessment form

	Not adequate (Score = 0)	Adequate (Score = 1)	Very good (Score = 2)	Score
Research design	Cross-sectional; Case study or other brief comment material;	Longitudinal (retrospective); Observational;	Longitudinal (prospective); Randomized Controlled Trial;	
Measurement of suicide-related outcome	Non-validated scale or other means of self-report (e.g. single question)	Hospital admission where intent has not been established; suicide items from a validated diagnostic/ mood rating scale (e.g. HDRS, BDI, SADS).	Clinical interview; validated suicide scale (e.g. SIS, SAS); hospital admission where intent has been established; death certificate/cause of death register	
Relationship between public health emergency and outcome	Poor quality evidence<br><br>No baseline; No control for confounders	Good quality of evidence.<br><br>Has baseline; No control for confounders	Very good quality evidence<br><br>Consideration for temporal relationship	
Study sample	Unrepresentative of target population	Representative of target population (at baseline)	Representative of target population (throughout data collection phase of study, consideration of attrition)	
Are the analyses sufficiently powered/is power relevant	No.<br><br>Insufficiently powered; Power is not reported	Yes<br><br>Power is reported and acceptable	-	
Total				

Note. Adapted from O'Connor and colleagues (2016).

Additional Publications on the COVID-19 Pandemic and Suicide-Related Outcomes

Preprint

Our complementary search of grey literature identified one preprint (Ammerman, Burke, Jacobucci, & McClure, 2020) (publication under peer-review; Table D). The results suggest that physical safety concerns and general distress due to COVID-19 were associated with increased past-month suicidal ideation, whereas changes to daily routine resulting from COVID-19 social distancing policies were associated with decreased past-month suicidal ideation. COVID-19-related experiences, such as general distress or the effects of social distancing policies, were associated with increased past-month attempted suicide (adjusted for age, gender, ethnicity, and past-month suicidal ideation). The investigators also reported that individuals who intentionally exposed themselves to COVID-19 were more likely to report past-month suicidal ideation and past-month suicide attempt(s).

Table D. Characteristics of included pre-print.

Study, Country, and study period	Population		Public Health Emergency		Suicide / Self-harm	Main findings	Quality assessment
	Sample / Source	Sex / Age	Exposure / Measures	Main public health responses	Outcomes / Measures		
Ammerman et al. (2020) [USA] Study period: 3-4 April 2020.	N = 907 General population via Amazon Mechanical Turk.	Gender: M = 511; F = 387. Age: Mean = 36.43 (18-74).	Exposure: COVID-19 pandemic. Measures: COVID-19-related experiences (e.g., exposure, distress about the disease, etc.) and intentional COVID-19 exposure.	Social distancing, stay-at-home orders.	Outcomes: Suicidal ideation, suicide attempt. Measures: Self-report items (two modified, face-valid items from the Self-Injurious Thoughts and Behaviors Interview by Nock et al. (2007).	Physical safety concerns and general distress due to COVID-19 were positively associated with past-month suicidal ideation ($r = 0.45$, $p < 0.01$). Daily routine impacts due to COVID-19 social distancing policies were negatively associated with past-month suicidal ideation. 45% of participants endorsing past-month suicidal thoughts reported their thoughts were explicitly linked to COVID-19. Individuals who intentionally exposed themselves to COVID-19 were more likely to report past-month suicidal ideation ($X^2(1) = 64.16$, $p < 0.001$, $V = 0.27$) and past-month suicide attempt(s) ($X^2(1) = 224.83$, $p < 0.001$, $V = 0.50$).	3

COVID-19 Case Studies

Our search also identified a small number of case reports whose findings do not yet appear to have been replicated in well-designed trials but are nevertheless included here for completeness. These reports document additional potential associations between the COVID-19 pandemic and both suicide deaths and suicide attempts.

Four unique publications presenting ten cases of suicide-related outcomes in the context of epidemics were identified. One such report in India (Goyal, Chauhan, Chhikara, Gupta, & Singh, 2020) found that an individual took his own life after being informed by his doctor that he had contracted a viral illness, which he wrongly associated with COVID-19. In another report by Griffiths and Mamun (2020), a series of six cases (four from India, one from the Bangladesh, and one from the USA) illustrated suicidal behaviour among couples and suicide pacts from the press. The cases described were retrieved from the media, and involved couples who attempted or died by suicide in contexts associated with the COVID-19 pandemic, such as economic recession, psychological suffering, and movement restriction. The authors described some of the motivating factors for COVID-19-related suicides, including fear of infection, financial constraints resulting from economic recession associated with national lockdowns, harassment or victimization by others for having (or being thought to have) COVID-19, and the stress of being in isolation or quarantine.

In another report, Mamun and Griffiths (2020) described the case of a Bangladeshi man who thought he was infected with COVID-19 and took his own life after suffering severe prejudice and social avoidance by his village members. Two other cases of emergency department presentation due to attempted suicide in India were described by Sahoo and colleagues (2020), both related to apprehension of developing COVID-19, information overload, and psychological distress (anxiety and depressive symptoms). These reports highlight the role of the media (social media platforms, newspapers, radio/telecommunication services) in exacerbating fears around infection and a sense of panic among vulnerable populations.

References

Ammerman, B. A., Burke, T. A., Jacobucci, R., & McClure, K. (2020). Preliminary Investigation of the Association Between COVID-19 and Suicidal Thoughts and Behaviors in the U.S. *PsyArXiv Preprint*. https://doi.org/10.31234/osf.io/68djp

Chan, S. M. S., Chiu, F. K. H., Lam, C. W. L., Leung, P. Y. V., & Conwell, Y. (2006). Elderly suicide and the 2003 SARS epidemic in Hong Kong. *International Journal of Geriatric Psychiatry*, *21*(2), 113–118. https://doi.org/10.1002/gps.1432

Cheung, Y. T., Chau, P. H., & Yip, P. S. F. F. (2008). A revisit on older adults suicides and Severe Acute Respiratory Syndrome (SARS) epidemic in Hong Kong. *International Journal of Geriatric Psychiatry*, *23*(12), 1231–1238. https://doi.org/10.1002/gps.2056

Falster, K., Choi, J. Y., Donovan, B., Duncombe, C., Mulhall, B., Sowden, D., … on behalf of the Australian HIV Observational Database, the T. A. H. I. V. O. D. (2009). AIDS-related and non-AIDS-related mortality in the Asia-Pacific region in the era of combination antiretroviral treatment. *AIDS*, *23*(17), 2323–2336. https://doi.org/10.1097/QAD.0b013e328331910c

Goyal, K., Chauhan, P., Chhikara, K., Gupta, P., & Singh, M. P. (2020). Fear of COVID 2019: First suicidal case in India ! *Asian Journal of Psychiatry*, *49*, 101989. https://doi.org/10.1016/j.ajp.2020.101989

Griffiths, M. D., & Mamun, M. A. (2020). COVID-19 suicidal behavior among couples and suicide pacts: Case study evidence from press reports. *Psychiatry Research*, *289*, 113105. https://doi.org/10.1016/j.psychres.2020.113105

Honigsbaum, M. (2010). The great dread: Cultural and psychological impacts and responses to the “Russian” influenza in the united kingdom, 1889-1893. *Social History of Medicine*, *23*(2), 299–319. https://doi.org/10.1093/shm/hkq011

Huang, C.-C., Yen, D. H.-T., Huang, H.-H., Kao, W.-F., Wang, L.-M., Huang, C.-I., & Lee, C.-H. (2005). Impact of severe acute respiratory syndrome (SARS) outbreaks on the use of emergency department medical resources. *Journal of the Chinese Medical Association*, *68*(6), 254–259. https://doi.org/10.1016/S1726-4901(09)70146-7

Keita, M. M., Taverne, B., Savané, S. S., March, L., Doukoure, M., Sow, M. S., … Delaporte, E. (2017).

Depressive symptoms among survivors of Ebola virus disease in Conakry (Guinea): Preliminary results of the PostEboGui cohort. *BMC Psychiatry*, *17*(1), 127. https://doi.org/10.1186/s12888-017-1280-8

Landis, J. R., & Koch, G. G. (1977). The Measurement of Observer Agreement for Categorical Data. *Biometrics*, *33*(1), 159–174. https://doi.org/10.2307/2529310

Mamun, M. A., & Griffiths, M. D. (2020). First COVID-19 suicide case in Bangladesh due to fear of COVID-19 and xenophobia: Possible suicide prevention strategies. *Asian Journal of Psychiatry*, *51*, 102073. https://doi.org/10.1016/j.ajp.2020.102073

O'Connor, D. B., Ferguson, E., Green, J. A., O'Carroll, R. E., & O'Connor, R. C. (2016). Cortisol levels and suicidal behavior: A meta-analysis. *Psychoneuroendocrinology*, *63*, 370–379. https://doi.org/10.1016/j.psyneuen.2015.10.011

Okusaga, O., Yolken, R. H., Langenberg, P., Lapidus, M., Arling, T. A., Dickerson, F. B., … Postolache, T. T. (2011). Association of seropositivity for influenza and coronaviruses with history of mood disorders and suicide attempts. *Journal of Affective Disorders*, *130*(1–2), 220–225. https://doi.org/10.1016/j.jad.2010.09.029

Sahoo, S., Rani, S., Parveen, S., Pal Singh, A., Mehra, A., Chakrabarti, S., … Tandup, C. (2020). Self-harm and COVID-19 Pandemic: An emerging concern – A report of 2 cases from India. *Asian Journal of Psychiatry*, *51*, 102104. https://doi.org/10.1016/j.ajp.2020.102104

Wasserman, I. M. (1992). The Impact of Epidemic, War, Prohibition and Media on Suicide: United States, 1910–1920. *Suicide and Life-Threatening Behavior*, *22*(2), 240–254. https://doi.org/10.1111/j.1943-278X.1992.tb00231.x

Yip, P. S. F. F., Cheung, Y. T., Chau, P. H., & Law, Y. W. (2010). The impact of epidemic outbreak: The case of severe acute respiratory syndrome (SARS) and suicide among older adults in Hong Kong. *Crisis*, *31*(2), 86–92. https://doi.org/10.1027/0227-5910/a000015
